# The Neural Basis of Metaphor Comprehension: Evidence from Left Hemisphere Degeneration

**DOI:** 10.1162/nol_a_00022

**Published:** 2020-10-01

**Authors:** Nathaniel Klooster, Marguerite McQuire, Murray Grossman, Corey McMillan, Anjan Chatterjee, Eileen Cardillo

**Affiliations:** Department of Neurology, Perelman School of Medicine, University of Pennsylvania, Philadelphia, PA, USA; Penn Frontotemporal Degeneration Center, University of Pennsylvania, Philadelphia, PA, USA; Department of Neurology, Perelman School of Medicine, University of Pennsylvania, Philadelphia, PA, USA; Department of Neurology, Perelman School of Medicine, University of Pennsylvania, Philadelphia, PA, USA; Penn Frontotemporal Degeneration Center, University of Pennsylvania, Philadelphia, PA, USA; Department of Neurology, Perelman School of Medicine, University of Pennsylvania, Philadelphia, PA, USA; Penn Frontotemporal Degeneration Center, University of Pennsylvania, Philadelphia, PA, USA; Department of Neurology, Perelman School of Medicine, University of Pennsylvania, Philadelphia, PA, USA; Moss Rehabilitation Research Institute, Elkins Park, PA, USA; Penn Center for Neuroaesthetics, University of Pennsylvania, Philadelphia, PA, USA; Department of Neurology, Perelman School of Medicine, University of Pennsylvania, Philadelphia, PA, USA; Penn Center for Neuroaesthetics, University of Pennsylvania, Philadelphia, PA, USA

**Keywords:** figurative language, neurodegeneration, left hemisphere, LpMTG, LIFG

## Abstract

Despite the ubiquity of metaphor in cognition and communication, it is absent from standard clinical assessments of language, and the neural systems that support metaphor processing are debated. Previous research shows that patients with focal brain lesions can display selective impairments in processing metaphor, suggesting that figurative language abilities may be disproportionately vulnerable to brain injury. We hypothesized that metaphor processing is especially vulnerable to neurodegenerative disease, and that the left hemisphere is critical for normal metaphor processing. To evaluate these hypotheses, we tested metaphor comprehension in patients with left-hemisphere neurodegeneration, and in demographically matched healthy comparison participants. Stimuli consisted of moderately familiar metaphors and closely matched literal sentences sharing the same source term (e.g., *The interview was a painful crawl* / *The infant’s motion was a crawl*). Written sentences were presented, followed by four modifier-noun answer choices (one target and three foils). Healthy controls, though reliably better at literal than metaphor trials, comprehended both sentence conditions well. By contrast, participants with left-hemisphere neurodegeneration performed disproportionately poorly on metaphor comprehension. Anatomical analyses show relationships between metaphor accuracy and patient atrophy in the left middle and superior temporal gyri, and the left inferior frontal gyrus, areas that have been implicated in supporting metaphor comprehension in previous imaging research. The behavioral results also suggest deficits of metaphor comprehension may be a sensitive measure of cognitive dysfunction in some forms of neurodegenerative disease.

## INTRODUCTION

Metaphor, the description of one idea in terms of another, plays an important role in cognition and communication. The use of metaphor is pervasive, accounting for more than 13% of words in written discourse and more than 6% of spoken language (Steen et al., 2010). Metaphor strongly influences people’s thinking in fundamental ways. The metaphors of “shaping” or “framing” are often used to describe the influence of metaphor on thinking (e.g., Lakoff & Johnson, 1980). Meta-analyses show that metaphorical language is more persuasive than comparable literal language (Sopory & Dillard, 2002; Van Stee, 2018). The presence or absence of metaphor when describing an issue affects how people think about topics such as cancer (e.g., Gibbs & Franks, 2002; Hauser & Schwarz, 2015), crime (Thibodeau & Boroditsky, 2011, 2013), significant personal relationships (Lee & Schwarz, 2014; Robins & Mayer, 2000), the acceptability of cognitive enhancement (Conrad, Humphries, & Chatterjee, 2019), the urgency of climate change (Flusberg, Matlock, & Thibodeau, 2017; Nerlich & Jaspal, 2012), and the brilliance of an idea (Elmore & Luna-Lucero, 2017).

Beyond influencing how people think, metaphor can affect how people act. Metaphor can drive attention (Matlock, 2004), affecting what information is attended to in social environments (Bowes & Katz, 2015). The choice of metaphor can affect how communities choose to handle public health issues (Barry, Brescoll, Brownell, & Schlesinger, 2009) and how patients are routed through health care facilities (Hilligoss, 2014). Because metaphorical language can be persuasive and can influence behavior, metaphors are widely used in didactic contexts (see the review in Saban, 2006), and in the teaching of medicine (Van Rijn-Van Tongeren, 1997), and in science specifically (Beger & Jäkel, 2015). Metaphor use is pervasive, it frames thinking, and it affects human behavior.

The neural bases of metaphor are debated. Early studies of metaphor comprehension focused on possible hemispheric differences, with some highlighting a privileged role for the right hemisphere (Bottini et al., 1994; Brownell, Simpson, Bihrle, Potter, & Gardner, 1990; Winner & Gardner, 1977). The right hemisphere hypothesis for metaphor argues that the right hemisphere plays a specific and necessary role in supporting the ability to understand metaphor. The right hemisphere is thought to be especially important in the comprehension of novel metaphors (Bohrn, Altmann, & Jacobs, 2012; Mashal & Faust, 2009; Mashal, Faust, & Hendler, 2005).

Over time, however, the role of the left hemisphere in metaphor comprehension has begun to be considered more seriously. Functional imaging studies indicate left hemisphere regions support metaphor comprehension in addition to right hemisphere regions (Bambini, Gentili, Ricciardi, Bertinetto, & Pietrini 2011; Cardillo, Watson, Schmidt, Kranjec, & Chatterjee, 2012; Chen, Widick, & Chatterjee, 2008; Lee & Dapretto, 2006; Obert et al., 2014; Schmidt & Seger, 2009; Yang, Fuller, Khodaparast, & Krawczyk, 2010), and sometimes perhaps exclusively (Diaz, Barrett, & Hogstrom, 2011; Rapp, Leube, Erb, Grodd, & Kircher, 2004, 2007). Meta-analyses of the functional imaging literature also suggest that metaphor comprehension is a bilaterally mediated process (Bohrn et al., 2012; Rapp, Mutschler, & Erb, 2012; Reyes-Aguilar, Valles-Capetillo,& Giordano, 2018; Yang, 2014). These studies indicate that the bilateral inferior frontal gyri and the left posterior middle temporal gyrus (LpMTG) specifically are activated during metaphor comprehension.

Neuroimaging studies have their inferential limitations. They are limited in making claims of a causal role for brain regions underlying a cognitive function. Patient studies, while more difficult to conduct, offer an important constraint on theorizing based solely on imaging studies (Fellows et al., 2005). Studies with focal lesion patients find evidence of patients with selective metaphor deficits despite normal literal sentence comprehension following left-sided damage (Cardillo, McQuire, & Chatterjee, 2018; Gagnon, Goulet, Giroux, & Joanette, 2003; Ianni, Cardillo, McQuire, & Chatterjee, 2014; Tompkins, 1990). These studies implicate the left frontal and posterior temporal cortices in metaphor comprehension (Cardillo et al., 2018; Zaidel, Kasher, Soroker, & Batori, 2002). The traditional view that the right hemisphere plays a privileged role in metaphor comprehension is increasingly difficult to justify, though it remains a staple of clinical and basic neuroscience teaching.

The neural bases for metaphor comprehension are relevant to models of language processing, but also for adequately addressing the therapeutic needs of clinical populations. Studies of diverse patient populations indicate metaphor processing is frequently impacted by brain injury or disease. Following a traumatic brain injury, patients displayed disruptions with metaphor processing (Yang et al., 2010). Studies also report impairments in metaphor comprehension in Parkinson’s disease (Fernandino et al., 2013; Monetta & Pell, 2007) and Alzheimer’s disease (Amanzio, Geminiani, Leotta, & Cappa, 2008; Papagno, 2001; Roncero & de Almeida, 2014; Winner & Gardner, 1977). Figurative language abilities more broadly are impaired in mild cognitive impairment (Cardoso, Silva, Maroco, de Mendonça, & Guerreiro, 2014) and in Alzheimer’s disease (Papagno, Lucchelli, Muggia, & Rizzo, 2003). These studies raise the possibility that metaphor comprehension is an especially fragile linguistic ability. In contrast to stable, focal lesions, neurodegenerative diseases have neural consequences and cognitive impairments that change gradually over time. Metaphor processing is a complex cognitive ability that requires contributions from many subdomains of cognition. Degenerative damage in distinct neural systems may lead to a common cognitive impairment. For instance, the effects of small decrements in multiple aspects of cognition could aggregate to a failure in metaphor comprehension. The complexity of metaphor processing and its resulting fragility in the face of distributed brain damage raise the possibility that metaphor comprehension is a sensitive measure of cognitive dysfunction in neurodegenerative disease.

### The Current Study

We hypothesized that metaphor processing relies on an intact left hemisphere and that metaphor comprehension is vulnerable to neurodegenerative disease. To evaluate these hypotheses, we tested metaphor comprehension with a rigorously controlled task in a group of patients with left hemisphere neurodegeneration (LHND), and in demographically matched healthy control (HC) participants. While many patient studies of metaphor processing are strictly behavioral, the current study related behavior to structural MRI. Patients first underwent structural MRI scans and later completed the metaphor task. Task performance was related to cortical thickness values. We predicted that patients would be especially impaired on metaphor trials (compared to HCs and compared to their own performance on literal trials) and that these impairments would be associated with patterns of left temporal lobe atrophy in the patient group. Based on results of functional imaging studies of metaphor processing using stimuli from the same set as used here (Cardillo et al., 2012), we assessed whether patterns of atrophy in three regions of interest (ROI) frequently engaged by metaphor tasks—the left inferior frontal gyrus (LIFG) , the right inferior frontal gyrus (RIFG), and the LpMTG—would relate to metaphor task performance.

## MATERIALS AND METHODS

### Metaphor Task

#### Target sentences

Stimuli included 32 matched metaphor–literal sentence pairs taken from the published stimuli sets of Cardillo, Schmidt, Kranjec, & Chatterjee, 2010; Cardillo, Watson, & Chatterjee, 2016). All 64 sentences were in the form “The *X* was a *Y*,” where Y was the shared word or phrase in the literal–metaphor match (hereafter, *source* term). The source term of the sentence, *Y*, was either an entity noun (e.g., *The relay was a sprint race* / *The math test was an intelligence race*) or an event noun (*The interruption was a loud knock* / *His emails were an insistent knock*). Source terms also always included sensorimotor features—half auditory (e.g., *knock*) and half motion (e.g., *race*). See Table 1 for examples.

**Table 1.** Stimuli examples

Sentence	Type	Example	Target	Foil 1	Foil 2	Foil 3
Metaphor	Entity-Auditory	Her chores were a sad tune.	gloomy routine	funeral hymn	playful exercise	prison system
	Entity-Motion	The summer romance was a merry-go-round.	dizzying delight	amusement ride	serious punishment	brick fireplace
	Event-Auditory	The cool breeze was a lullaby.	calming weather	reassuring melody	uncomfortable temperature	cracked basin
	Event-Motion	The prize money was a lift.	financial assistance	small elevator	economic burden	delicious apple
Literal	Entity-Auditory	The jingle was a happy tune.	catchy song	loose change	radio static	flower vase
	Entity-Motion	The construction was a new merry-go-round.	colorful carousel	real estate	moldy dungeon	grocery cart
	Event-Auditory	The child’s favorite was a lullaby.	soothing song	biological offspring	screaming match	cracked knuckles
	Event-Motion	The bed was a heavy lift.	weighty mattress	striped sofa	lightweight frame	full trashcan

Stochastic Optimization of Stimuli software (Armstrong, Watson, & Plaut, 2012) was used to ensure that metaphor and literal sentences were matched on many of the published norms for the items: the number of characters, words, and content words; and the average frequency and concreteness of their content words; as well as their valence (% positive), an online measure of semantic processing difficulty (valence judgment reaction time), and familiarity (all *p*’s > 0.09). Values for these variables were taken from published norms (Cardillo et al., 2010; Cardillo et al., 2016). Overall, metaphors were moderately familiar (4.6 / 7; 1 = *very unfamiliar*, 7 = *very familiar*) and were well-understood (Interpretability *M* = 0.91, *SD* = 0.09). Metaphors were less imageable than their literal counterparts (*p* < 0.01) and, as intended, more figurative (*p* < 0.01). These differences and equivalences held true whether collapsing across variables of noninterest (modality, source term), or whether distinguishing items further by these characteristics. For item properties, see Supplementary Table 2 in the online supporting information located at https://www.mitpressjournals.org/doi/suppl/10.1162/nol_a_00022.

#### Answer choices

Each target sentence was accompanied by four possible answers, a correct target and three foils. Each answer choice consisted of a modifier (adjective or noun) and a noun. Foils for the metaphor stimuli (Table 1) were (1) the literal meaning of the sentence, (2) the opposite of the metaphorical meaning of the sentence, and (3) an unrelated answer. Foils for the literal sentences were (1) a category associate of the agent of the sentence not implied by the sentence, (2) the opposite of the literal meaning of the sentence, and (3) an unrelated answer. In this way, answer choices were designed to be informative of the nature of comprehension difficulty on incorrect trials. Answer choices were matched on average frequency, concreteness, and valence. For full materials used in this study see Supplementary Table 1 in the online supporting information.

#### Task procedure

For both groups, stimuli were presented visually on a laptop using E-Prime 2.0 software. On every trial, a sentence was presented at the top of the screen. When the participant indicated they were done reading the target sentence, the sentence remained on the screen and answer choices were presented below it, randomized to one of four quadrants in the lower half of the screen. Participants were instructed to choose the answer that best matched the meaning of the sentence and to guess if unsure.

Patients read the sentences and indicated to a researcher (orally or by pointing) which answer they thought best matched the meaning of the sentence. To limit demands on working memory for patients, the research assistant recorded the patient’s answer and advanced the trial. HCs controlled the testing laptop and made their responses without assistance.

### Piloting Process

Ten adults free of neurological disease or brain injury and with overall cognition and verbal intelligence within the normal range (*Mean* = 27.9 ± 2.0, Mini-Mental State Examination [MMSE; Folstein, Folstein, & McHugh, 1975]; Mean = 113.6 ± 10.1, American National Adult Reading Test [AMNART; Nelson & O’Connell, 1978]) were recruited from a database of healthy older adults to serve as pilot participants for the metaphor and literal multiple-choice stimuli. Participants were paid $15/hr and gave informed consent for their participation in accordance with procedures of the University of Pennsylvania Institutional Review Board (protocol #806447). Pilot participants were native English speakers and were matched to the patient group on age (*M* = 69.5 ± 7.5) and education (*M* = 15.3 ± 3.0).

Pilot participants completed the task as described above. Item accuracy analysis revealed seven of the 64 items for which the correct answer was selected ≤60% of the time. Based on the foils most commonly chosen for these items, target answers and/or foils were revised to disambiguate the answer choices. Target sentences were not altered. Patients and a new group of HCs were tested on these revised stimuli.

### Participants

#### Healthy comparison participants

Nineteen HCs were recruited from a database of healthy older adults to participate in the study. HCs were paid $15/hr and gave informed consent in accordance with procedures of the University of Pennsylvania Institutional Review Board (protocol #806447). HCs were native English speakers, matched to the patient group on age (*M* = 65.2 ± 10.2) and education (*M* = 15.4 ± 2.6). Neuropsychological testing confirmed they scored within the normal range on the MMSE (*M* = 28.8 ± 1.6), indicating normal cognition, and on the AMNART (*M* = 117.83 ± 8.20), indicating normal verbal intelligence.

#### Patients

Thirteen patients were recruited from a Frontotemporal Degeneration Center to participate in the study. All patients were diagnosed with logopenic-variant primary progressive aphasia (lvPPA) at the time of testing, according to established criteria (Gorno-Tempini et al., 2011), and confirmed through a consensus process. Since this initial diagnosis, on reassessment three were diagnosed with progressive supranuclear palsy, one with Alzheimer’s disease, one with behavioral-variant fronto-temporal dementia, and one with nonfluent PPA. These were clinical diagnoses based only on phenotype. The remaining patients retained a lvPPA diagnosis at the time of publication. Patients and HC participants were matched on age and education. Patients displayed mild impairment on the MMSE (*M* = 24.62, Tables 2 and 3) and as a group performed significantly worse than HCs (Table 2). Patients were paid $15/hr and gave informed consent in accordance with procedures of the University of Pennsylvania Institutional Review Board (protocol #806447).

**Table 2.** Demographics

Group	Age	Chronicity	Education	MMSE
LHND (*n* =13)	63.31 (± 6.9)	3.69 (± 1.49)	15.62 (± 2.9)	24.62 (± 4.5)**
HC (*n* = 19)	65.21 (± 10.2)		15.39 (± 2.6)	28.58 (± 1.6)**

*Note*. ** HC MMSE > LHND (*p* < 0.01). LHND = left-hemisphere neurodegeneration, HC = healthy control, MMSE = Mini-Mental State Examination.

**Table 3.** Patient neuropsychological profile

Subject	LHND1	LHND2	LHND3	LHND4	LHND5	LHND6	LHND7	LHND8	LHND9	LHND10	LHND11	LHND12	LHND13	Mean
PVLT Recall	9	8	3	5	4	4	2	0	8	3	8	8	0	4.77
PPT Words	24	25	25	25	21	16	25	23	23	25	13	22	24	22.38
PPT Pictures	26	26	24	25	23	19	26	22	24	26	24	24	24	24.10
Naming	93.33	86.67	90	40	60	36.67	81.25	18.75	93.33	93.75	96.88	78.13	–	57.9
Animal Fluency	19	19	10	11	1	6	11	5	13	10	16	7	6	10.31
“f” fluency	13	15	7	7	3	10	7	5	4	17	9	4	4	8.08
Trails A Time	31	35	46	45	–	89	37	30	48	130	34	54	66	126.23
Trails B Time	80	72	300	130	–	282	211	166	228	300	182	181	–	193.82
PVLT1	5	4	1	1	3	1	1	0	4	4	4	5	0	2.54
Digit Span Forward	3	3	5	5	5	5	1	2	2	4	4	10	2	3.92
Digit Span Backward	5	5	4	3	0	6	2	2	3	4	4	7	1	3.54
MMSE	29	27	23	28	25	19	21	26	28	20	15	29	30	24.61

*Note*. LHND = left-hemisphere neurodegeneration, PVLT = Philadelphia Verbal Learning Test (Libon, Mattson, Glosser, & Kaplan, 1996), PPT = Pyramids and Palm Trees (Howard & Patterson, 1992), MMSE = Mini-Mental State Exam.

### Anatomical Methods

#### T1 Whole-brain imaging

High-resolution T1-weighted MPRAGE structural scans were acquired for all but one patient (excluded due to claustrophobia and anxiety even following sedation) and 35 healthy controls comparable to the patient group (Age: *M* = 67.37, *p* < 0.15; Education: *M* = 16.03, *p* < 0.65; 10 males). MRI data was acquired on a 3T Siemens Tim Trio scanner with an 8-channel head coil, with T = 1,620 ms, T = 3.09 ms, flip angle = 15°, 192 × 256 matrix, and 1 mm^3^ voxels. T1-weighted MRI images were then preprocessed to compute cortical thickness using Advanced Normalization Tools (ANTs; Tustison et al., 2014). Briefly, each individual dataset was deformed using a symmetric and diffeomorphic registration routine using ANTs to register each volume to a standard local template space in a canonical stereotactic coordinate system. ANTs provide a highly accurate registration routine using symmetric and topology-preserving diffeomorphic deformations to minimize bias toward the reference space and to capture the deformation necessary to aggregate images in a common space. Then, we used N4 bias correction to minimize heterogeneity (Tustison et al., 2010) and the ANTs Atropos tool to segment images into six tissue classes (cortex, white matter, cerebrospinal fluid, subcortical grey structures, brainstem, and cerebellum) using template-based priors, and to generate probability maps of each tissue. Voxel-wise cortical thickness was measured in millimeters (mm) from the pial surface and then transformed into Montreal Neurological Institute (MNI) space, smoothed using a 2 sigma full-width half-maximum Gaussian kernel, and downsampled to 2 mm isotropic voxels.

We conducted several types of anatomic analyses. The first targeted specific ROIs based on activation patterns in previous fMRI studies of metaphor comprehension. The second examined single-subject atrophy patterns in a subgroup of patients who show metaphor impairment. Exploratory supplemental analyses probed brain-behavior relationships of task performance within areas of degeneration as identified by a group mask.

**Figure 1.**
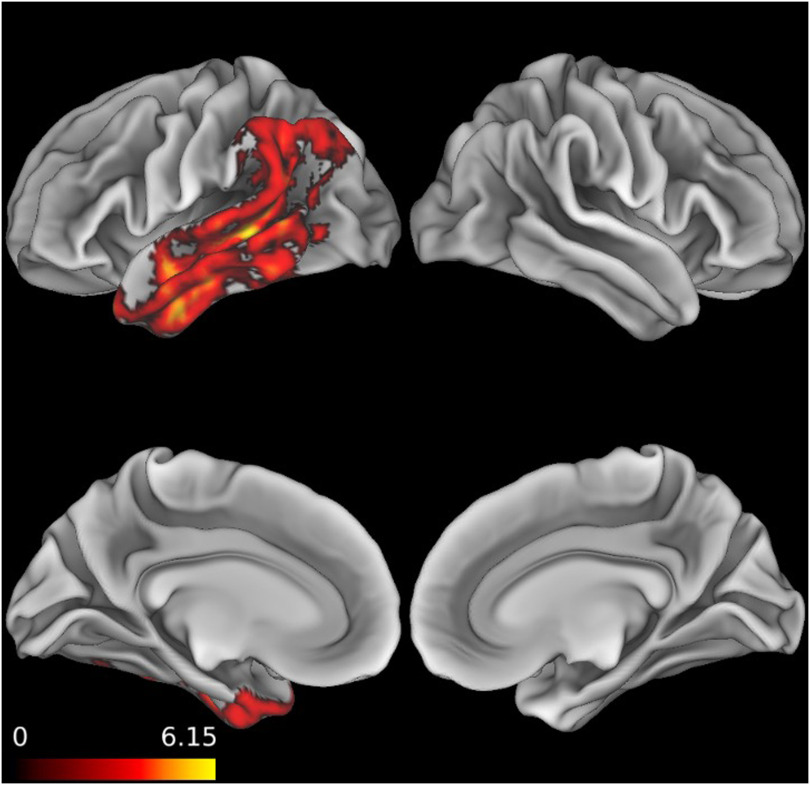
Patient whole brain atrophy. Pattern of cortical thinning in patients compared to healthy comparisons (significant at *p* < 0.005, family-wise error corrected with threshold-free cluster enhancement).

To characterize brain areas where the patients as a group displayed significant neural degeneration relative to matched controls, a patient atrophy mask (Figure 1 and Supplementary Table 3 in the online supporting information) was calculated through nonparametric permutation-based analyses with threshold-free cluster enhancement (TFCE; Smith & Nichols, 2009) with the randomize tool in FSL (http://fsl.fmrib.ox.ac.uk/fsl/fslwiki). Cortical thickness was compared across groups. To depict atrophic areas that show maximal overlap in the group, clusters that meet a conservative threshold of *p* < 0.005 (family-wise error corrected with TFCE), and contain a minimum of 200 adjacent voxels are reported.

When a more liberal threshold of *p* < 0.05 is applied, areas of variability driven by smaller subsets of participants are evident (Supplementary Figure 1 in the online supporting information). Patients varied widely in the extent of atrophy present in regions outside the left temporal lobe.

To specifically probe ROIs implicated as critical nodes in the metaphor comprehension network, individual variability in patient performance was related to variability in cortical thickness in three ROIs: the LIFG, the RIFG, and the LpTMG temporal gyrus. ROIs were generated by creating 10 mm radius spheres around the peak coordinates in MNI space reported for each of these clusters in an fMRI study of metaphor comprehension using similar stimuli (LIFG = −50, 29, −1; RIFG = 50, 26, 5; LpMTG = −62, −50, −9; Cardillo et al., 2012). Performance across literal and metaphor conditions was related to cortical thickness in these ROIs while controlling for age, sex, and years of education.

To characterize individual subject patterns of reduced cortical thickness in three individuals from the metaphor-impaired subgroup (see Neuroanatomical Results), individualized heatmaps of *Z*-transformed cortical thickness relative to 156 demographically comparable healthy adults with a self-reported negative psychiatric and neurological history were generated. These HCs were additionally screened as cognitively normal using a >27 (out of 30) score on the MMSE. To generate these heatmaps, the mean and *SD* for each voxel in template space for the control cohort were calculated. Then, *Z* scores of each patient’s whole brain cortical thickness map relative to the HC means and *SD*s were generated. Individual heatmaps were then masked by the patient atrophy mask and can be interpreted as an LHND-specific *Z* map of age, sex, and education appropriate patterns of reduced cortical thickness.

Supplemental exploratory analyses relate task performance to cortical atrophy in the patient group as a whole. The randomize tool in the FMRIB Software Library was used to run regression analyses between the behavior of interest and patient cortical thickness values. Nonparametric permutations (*n* = 10,000) were run for each score of interest. Clusters that met a height threshold of *p* < 0.05 uncorrected with TFCE and a minimum of 25 adjacent voxels are reported. A 25-voxel threshold was chosen.

### Statistical Methods

Linear mixed-effects models (LMEMs) were used to analyze the influence of variables of interest on metaphor task performance. The lme4 package (Bates, Maechler, Bolker, & Walker, 2015) in R (Version 3.3.1) was used for the following analyses. A theoretically motivated model was built for each analysis.

## RESULTS

### Behavioral Results

Analysis of HC performance revealed 1 of the 19 participants performed more than 2.5 *SDs* below the group mean (overall accuracy = 42.2% correct; 46.9% literal, 37.5% metaphor). This participant’s data was removed from further analysis.

HC participants responded to two of the items with low accuracy, indicating that these items were statistical outliers (for both items, only 8/18 participants answered correctly placing these items more than 2.5 *SDs* below the other items’ mean accuracy). These two items were removed from further analysis. The inclusion of these items, or of the outlier participant described above, did not affect the patterns of results described below.

**Figure 2.**
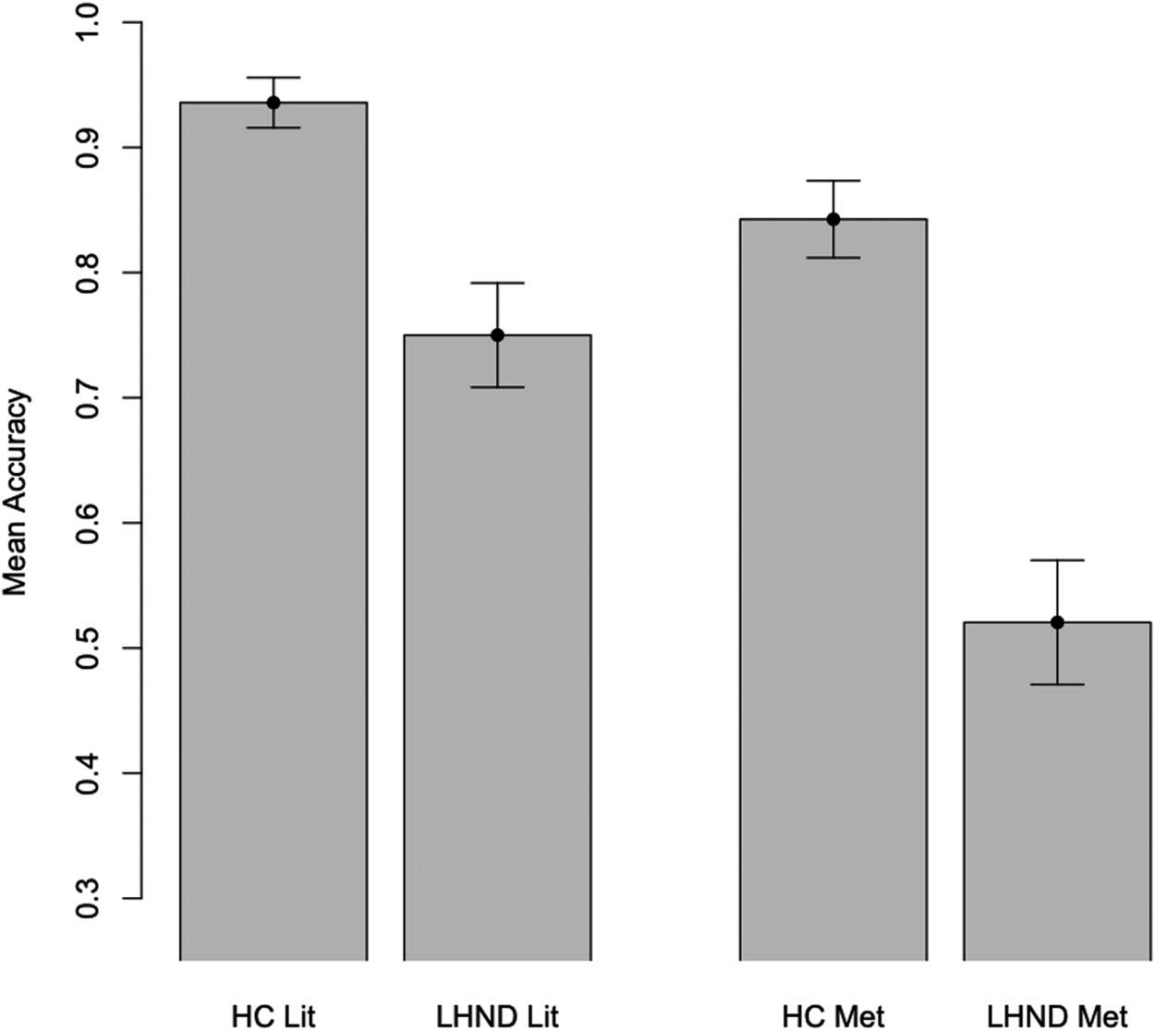
Metaphor task performance. Accuracy on literal (Lit) and metaphor (Met) trials by group showing significant effects of group (HC > LHND, *p* < 0.01), figurativeness (literal accuracy > metaphor accuracy, *p* < 0.05), and their interaction (with LHND especially impaired on metaphor accuracy, *p* < 0.05). LHND = left-hemisphere neurodegeneration, HC = healthy control.

To examine group differences, LMEMs were used to analyze the relationships between group (patients, HC), figurativeness (literal, metaphor), and accuracy. As random effects, participant and item were included as intercepts, with a by-subject random slope included for the effect of figurativeness and a by-group random slope included for item. These analyses (Tables 4 and 5 and Figure 2) revealed a fixed effect of figurativeness, with literal sentences receiving more accurate responses than metaphors (*p* < 0.05, Cohen’s *d* = 0.23), a fixed effect of group, with HCs outperforming LHND patients (*p* < 0.01, *d* = 0.48), and an interaction between group and figurativeness with the LHND group performing especially poorly on the metaphor stimuli (*p* < 0.05, *d* = 0.34). The difference between auditory (75.96%) and motion (79.55%) accuracy was not significant. There were no interactions of modality with group or figurativeness. There was no significant difference between event (80.00%) and entity (76.88%) accuracy, and there were no interactions of source with group or figurativeness.

**Table 4.** Percent accuracy by group and figurativeness

	Literal	Metaphor	Mean
LHND (*n* = 13)	75.0	50.51	63.90
HC (*n* = 18)	93.58	84.26	89.10
Mean	85.79	70.75	

*Note*. LHND = left-hemisphere neurodegeneration, HC = healthy control.

**Table 5.** Fixed effects

Parameter	Estimate	Standard Error	*t* value
Intercept	0.533194	0.125328	4.254***
Group (LHND)	−0.189489	0.054223	−3.495**
Education	0.026019	0.007747	3.359**
Figurativeness (Metaphor)	−0.093171	0.040388	−2.307*
Group (LHND) × Figurativeness (Metaphor)	−0.136316	0.057948	−2.352*

*Note*. * *p* < 0.05; ** *p* < 0.01; *** *p* < 0.001. LHND = left-hemisphere neurodegeneration.

#### Error analysis

To illuminate the nature of comprehension failures, the proportion of each foil type selected on incorrect trials was calculated. For metaphor trials, HCs (87%) and LHND (78%) showed a strong literal bias, most often choosing the foil that provided the literal meaning to the target metaphor rather than the correct metaphorical meaning. For literal trials, HCs (48%, 48%) and LHND (46% vs. 38%) showed an even split between the semantic associate and the opposite meaning foils.

### Patients

#### Single case analyses

To examine different patterns of impaired comprehension, individual patient performance was compared to the HC group using LMEMs. Subgroups based on behavior were then examined for different patterns of atrophy. Accuracy was modeled as a function of group identity (patient or HC), figurativeness (literal vs. metaphor trials), and education, and an interaction between figurativeness and group, with the random effects of subject and item. The Holm method was used to correct for multiple comparisons.

These analyses revealed that four patients (LHND2, LHND3, LHND4, LHND11) performed indistinguishably from HCs overall and comprehended the literal and metaphor stimuli alike (*p*’s > 0.05). Five patients displayed a general sentence-comprehension impairment (LHND5, LHND6, LHND8, LHND10, LHND13), with impaired performance on the task overall and on the literal stimuli (all *p*’s < 0.0001). For four patients, there was an interaction between group (patient or HC) and figurativeness (all *p*’s < 0.05), showing evidence for a disproportionate metaphor impairment, with worse metaphor comprehension performance than predicted by their literal sentence performance (LHND1, LHND7, LHND9, LHND12; see Table 6). LHND7 could not complete scanning due to anxiety. While all were diagnosed with lvPPA at the time of testing, LHND7 was subsequently diagnosed with Alzheimer’s disease, while the other patients retained a diagnosis of lvPPA at the time of publication.

**Table 6.** Single cases with metaphor impairment

Patient	Literal (32 max)	Literal *t* value	Literal *p* value	Metaphor (30 max)	Metaphor *t* value	Metaphor *p* value	Cohen’s *d*
LHND1	30	0.03	0.488	17	−1.77	0.048	0.894
LHND7	30	0.03	0.488	17	−1.77	0.048	0.894
LHND9	26	−2.14	0.024	13	−2.62	0.009	0.909
LHND12	28	−1.05	0.154	16	−1.98	0.032	0.794

*Note*. *p*-values are one-tailed and compared to the control group’s performance using the Crawford-Howell test (Crawford & Howell, 1998) for case-control comparisons. They provide a point estimate of the abnormality of each patient’s score. Cohen’s *d* values are the effect sizes for the differences between conditions for each patient. LHND = left-hemisphere neurodegeneration.

#### Neuropsychological measures and performance

In the patient group, task performance was related to performance on standard neuropsychological assessments (Table 3). Item accuracy was modeled as a function of the fixed effects of figurativeness and education, with random intercepts of subject and item and a by-subject random slope for figurativeness. As the current study is not powered to consider all the neuropsychological tests’ influence on task performance in the same model, the neuropsychological tests were examined one at a time for their ability to predict task performance by evaluating whether their inclusion improved model fit.

These analyses revealed that while many tests were related to performance on the literal sentences, including MMSE (*p* < 0.05, *d* = 0.0695), reverse Digit Span (*p* < 0.05, *d* = 0.1505), Naming (*p* < 0.05, *d* = 0.2314), Animals (*p* < 0.005, *d* = 0.0790), Philadelphia Verbal Learning Test (Libon, Mattson, Glosser, & Kaplan, 1996) recall (*p* < 0.05, *d* = 0.1005), Pyramids and Palm Trees (PPT; Howard & Patterson, 1992) picture (*p* < 0.05, *d* = 0.1408), PPT word (*p* < 0.001, *d* = 0.1165), and Complex Figure Test copy (*p* < 0.01, *d* = 0.1141), only lexical fluency was related to metaphor accuracy (*p* < 0.01, *d* = 0.0570).

### Neuroanatomical Results

#### ROI analyses

Relationships between cortical thickness and literal and metaphor performance were investigated in three ROIs (LIFG, RIFG, LpMTG) motivated by previous fMRI results indicating that these regions are engaged in comprehension of metaphors from the same stimulus set as used in this study. Patient cortical thickness did not differ from that of HCs in the RIFG (*p* > 0.91, *d* = 0.036), while the LIFG showed a trend and a much larger effect size (*p* < 0.11, *d* = 0.591). Cortical thickness in HCs did not differ between the RIFG and the LIFG (*p* < 0.37), while there was a significant difference in these areas in the patient group (*t* = 6.48, *p* < 0.0001, *d* = 0.322), with the LIFG showing reduced thickness compared to the RIFG. No relationship was seen between overall performance or literal performance and integrity of these areas in the patients (*p*’s > 0.7). On metaphor comprehension, LIFG thickness was significantly associated with accuracy (*p* < 0.05, *d* = 0.368), while RIFG thickness showed a trend (*p* > 0.18).

**Figure 3.**
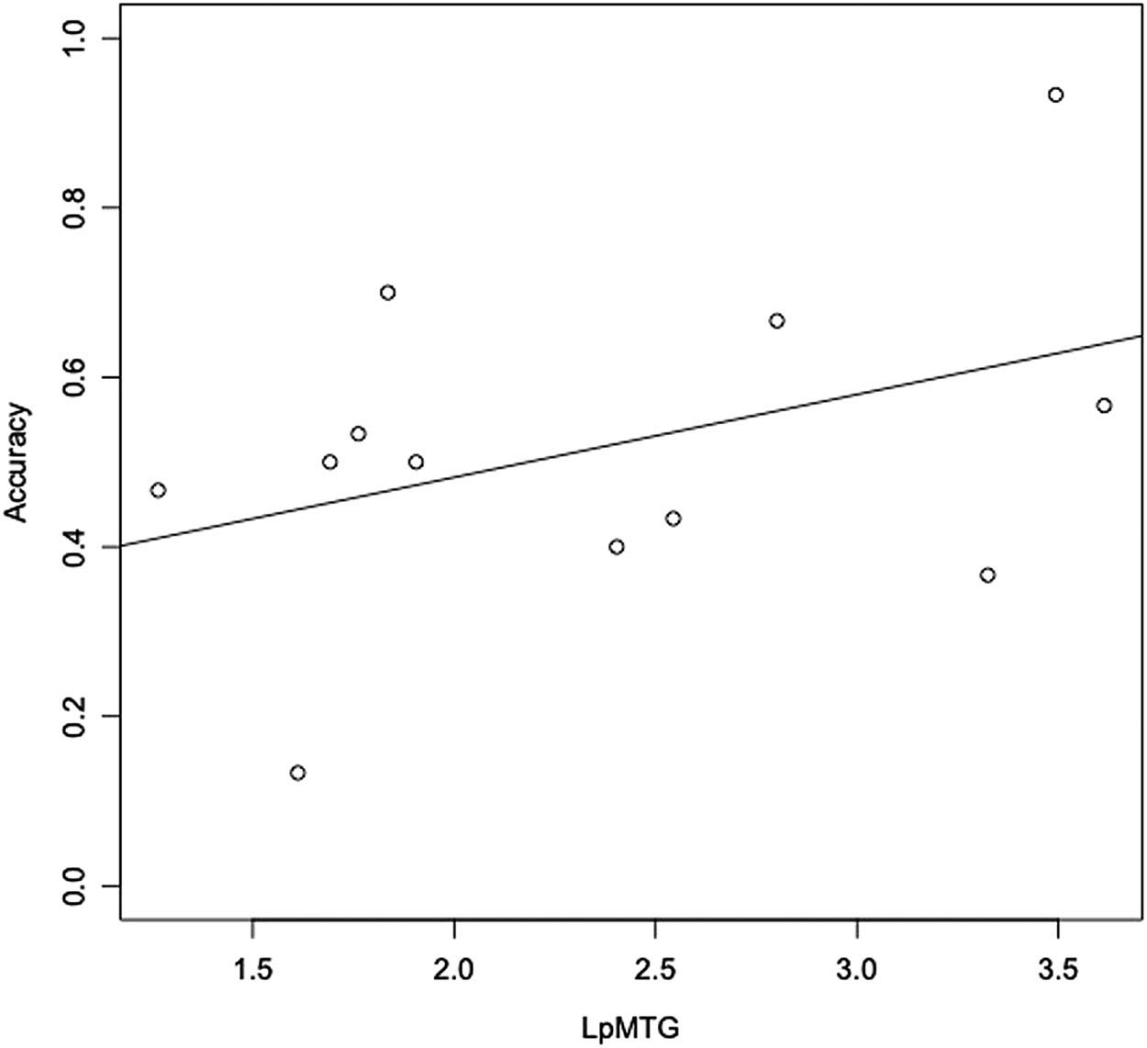
Metaphor trial accuracy in patients as a function of LpMTG cortical thickness (mm). LpMTG = left posterior middle temporal gyrus.

Patients showed significant atrophy relative to HCs in the LpMTG (*p* < 0.01, *d* = 1.174). For overall task performance, the LpMTG showed a trend toward being associated with accuracy (*p* < 0.098). Atrophy in the LpMTG did not relate to literal accuracy (*p* > 0.3). On metaphor trials (Figure 3), LpMTG thickness was significantly related to accuracy (*p* < 0.01, *d* = 0.730).

#### Single subject analyses

**Figure 4.**
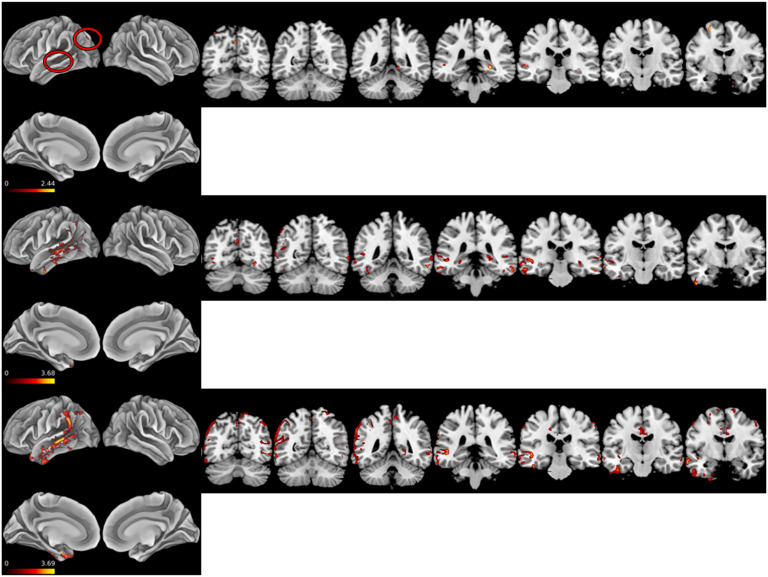
*Z* score heat maps for three patients with disproportionate metaphor impairment. Top: LHND1 Middle: LHND9. Bottom: LHND12. LHND = left-hemisphere neurodegeneration.

Figure 4 and Supplementary Table 3 in the online supporting information depict single subject atrophy heatmaps for the three participants with a disproportionate metaphor deficit and available neuroimaging.

#### Supplemental analyses: VBM within areas of degeneration

Exploratory voxel-based morphometry regressions were run relating patient performance on literal and metaphor trials to the cortical thickness values within their atrophy mask (Figure 1). For literal stimuli (Supplementary Figure 2 and Supplementary Table 4), atrophy in the superior temporal gyrus and the angular gyrus related to poorer accuracy. For metaphor stimuli (Supplementary Figure 3 and Supplementary Table 4), atrophy in the fusiform gyrus and the middle temporal gyrus related to poorer accuracy.

## DISCUSSION

Metaphor is used pervasively in communication and in cognition, shaping thought and influencing behavior. How the brain mediates comprehension of metaphor is debated. To investigate the neural bases of metaphor comprehension, we tested patients with neurodegenerative disease affecting the left hemisphere and a group of demographically matched HC participants on their ability to resolve the meaning of metaphoric and matched literal sentences. To summarize our results before discussing them in detail, our patients performed poorly on metaphor trials compared to HCs and compared to their own performance on literal trials. These results point to the important role that the left hemisphere plays in understanding metaphor. Thinning of LIFG, left middle temporal gyrus, and left superior temporal gyrus, areas implicated in previous work as supporting metaphor comprehension, was associated with poor metaphor performance in some of our patients here. The results also show that metaphor processing can be disproportionately impaired compared to literal sentence comprehension, suggesting that metaphor comprehension deficits could be a sensitive measure of cognitive change in neurodegenerative disease, revealing impairments before literal language is impacted.

The variability in previously proposed neural substrates for metaphor comprehension present in the literature may have risen from relative lack of control of psycholinguistic properties of sentences used to test brain-behavior relationships (Cardillo et al., 2010; Citron & Goldberg, 2014; Schmidt, Kranjec, Cardillo, & Chatterjee, 2010). By measuring and balancing literal and metaphor stimuli on various properties impacting comprehension difficulty—number of characters, words, and content words, average frequency and concreteness of content words, and familiarity, valence, and a measure of semantic processing speed of sentences—the current study attempted to avoid these difficulties and provide a more balanced contrast between literal and metaphor sentences.

The patients’ impaired metaphor task performance cannot be explained as following from general cognitive impairment. While analyses relating patient task performance to their neuropsychological test performance are exploratory given the relatively small sample size, patients’ neuropsychological profiles were mostly not associated with metaphor deficits. The patients’ performance on literal trials related to many neuropsychological test scores, including tests of overall cognition, working memory, episodic memory, and semantic memory. It makes sense that patients struggling to understand simple literal sentences would display impairments in multiple cognitive domains. The lack of clear relationships observed here between performance on metaphor trials and other aspects of cognition as measured through psychometric testing has been noted in other patient studies of metaphor (Amanzio et al., 2008). This observation affirms that traditional neuropsychological tests do not adequately capture metaphoric language deficits. We agree with the suggestion (Rapp & Wild, 2011) that nonliteral language should be included in standard neuropsychological assessment batteries.

The observation raises the question of why the patients struggle on metaphor trials. One possibility is that the inhibitory demands of metaphor comprehension are difficult for patients. To resolve the meaning of a metaphor, the literal sense of the sentence or features of the source term must be inhibited in order to identify the correct figurative sense of the word and sentence (Gernsbacher & Robertson, 1999; Papagno, 2001). The error analysis suggests that this inhibition is difficult for healthy participants. On incorrect trials, the literal sense was most often endorsed by HCs. For patients too, the literal foil was most often chosen on incorrect trials. The patients failed more often here than HCs, suggesting greater difficulty with the inhibitory demands of metaphor trials. A failure of inhibitory control also accords with the observation that lexical fluency—an index of executive function—was correlated in this population with metaphor comprehension accuracy. Successfully resolving the meaning of a metaphorical sentence also requires greater flexibility as features and properties of one domain are applied and compared to another. Cognitive flexibility is often diminished with general atrophy (Eslinger, Moore, Anderson, & Grossman, 2011; Kehagia, Barker, & Robbins, 2010; Swartz, Stuss, Gao, & Black, 2008) and this association may be contributing to the patients’ deficits.

Our results provide important evidence for left-hemisphere mediation of metaphor. In this study, patients with neurodegeneration restricted to the left hemisphere displayed deficits in understanding moderately familiar metaphors. Functional imaging evidence also implicates areas in the left hemisphere used by the healthy brain in metaphor comprehension (Bohrn et al., 2012; Rapp et al., 2012; Yang, 2014). The current study and other patient studies (Cardillo et al., 2018; Gagnon et al., 2003; Ianni et al., 2014; Tompkins, 1990) provide evidence for the importance of left-hemisphere regions for normal metaphor comprehension. Our anatomic analyses included a targeted ROI analysis, an analysis within areas of group degeneration, and a subgroup brain-behavior analysis of patients based on their patterns of performance. For the ROI analysis, metaphor comprehension related to thickness of LpMTG and LIFG.

The left temporal lobe and LIFG are linked to semantic ambiguity resolution in literal language (Zempleni, Renken, Hoeks, Hoogduin, & Stowe, 2007; Davis et al., 2007). When words have multiple senses, or when multiple concepts can resolve the meaning of an ambiguous sentence, these left hemisphere structures are implicated in successful meaning resolution. This ability shares similarities to the challenge of resolving the meaning of a metaphor, a sentence with multiple possible meanings to be resolved, and indeed similar brain regions are implicated in supporting these abilities.

The LpMTG and the LIFG were related to metaphor comprehension deficits, consistent with previous neuroimaging studies of metaphor (Cardillo et al., 2012; Chen et al., 2008; Lee & Dapretto, 2006; Schmidt & Seger, 2009; Yang et al., 2010) and suggesting an important role for these regions. While such a relationship was absent in RIFG, this may be because the current patient sample was not suitable for testing its role, as the patients show atrophy in LIFG but not RIFG. The LpMTG has been linked to demands of semantic processing more generally (e.g., Noonan, Jefferies, Visser, & Lambon Ralph, 2013).

The single-subject atrophy maps document the relationship between the left temporal lobe and metaphor trial accuracy broadly. All three show relationships between LpMTG integrity and metaphor trial performance. Interestingly, all three display a relationship between medial temporal lobe atrophy and metaphor performance. Metaphor task performance may be a sensitive measure of cognitive decline in neurodegenerative diseases that affect the medial temporal lobe such as Alzheimer’s disease.

When considering anatomy and neuropsychological profile and their relation to successful metaphor comprehension, there may be “many routes to failure.” As the network of brain regions that support metaphor processing is delineated, it is likely that disruptions to any nodes of this network, or the connections between them, could lead to impairment. Metaphor processing is a complex cognitive ability that depends on many subdomains of cognition including semantic memory, working memory, (semantic) executive demands, inhibition, abstract thinking, and cognitive flexibility. It is possible that subtle disruptions to any of these subdomains can lead to impaired metaphor comprehension.

Limitations of the current study include the sample sizes of the participant groups. The current results should be replicated with larger groups of participants. A priori power analyses were not completed. We tested as many patients as possible. Given the relatively small sample size in the current study and the difficulty of recruiting such patients, we elected to limit our ROI analyses to a few strongly motivated areas. Analyses relating patient task performance to neuropsychological assessment should be examined in larger populations. HC participants were closely matched to the patients demographically, and their behavioral data was normally distributed, but a larger comparison group would strengthen the confidence in our findings. Despite these limitations, robust group differences were observed, and strong relationships were detailed between anatomy and metaphor task performance.

The current study provides evidence that metaphor processing can be disproportionately impaired compared to literal sentence comprehension. The evaluation of metaphor processing may provide a more sensitive assessment of the earliest cognitive changes in neurodegenerative disease. Tests of metaphor may reveal impairments before literal language is impacted. While the cognitive consequences of focal lesions are observed rapidly, neurodegenerative disease processes are progressive and are often quite subtle in the earliest stages. More sensitive measures of cognitive change are needed as screening instruments and as outcome measures in the study of neurodegenerative disease. Metaphor processing, with its distributed neural support and cognitive complexity may be sensitive to early anatomical and cognitive changes.

In conclusion, we show that patients can display a disproportionate deficit in metaphor comprehension, compared to their own performance on literal sentence comprehension and to HC performance. The left hemisphere plays an important role in metaphor processing.

## FUNDING INFORMATION

Anjan Chatterjee, NIH, Award ID: R01DC012511. Nathaniel Bloem Klooster, NIH, Award ID: T32 HD071844.

## AUTHOR CONTRIBUTIONS

Nathaniel Klooster analyzed the data, created figures, and wrote the manuscript. Marguerite McQuire designed the study, created stimuli, and collected data. Murray Grossman oversaw experimental and neuropsychological data collection and contributed to manuscript writing. Corey McMillan created figures and contributed to manuscript writing. Anjan Chatterjee designed the study and contributed to manuscript writing. Eileen Cardillo designed the study, created stimuli, and contributed to manuscript writing.
