# Supplementary figures and images for: The Neural Basis of Metaphor Comprehension: Evidence from Left Hemisphere Degeneration
Source: Neurobiol Lang (Camb). 2020 Oct 1;1(4):474–91. doi: 10.1162/nol_a_00022 (PMC10158586; doi:10.1162/nol_a_00022)

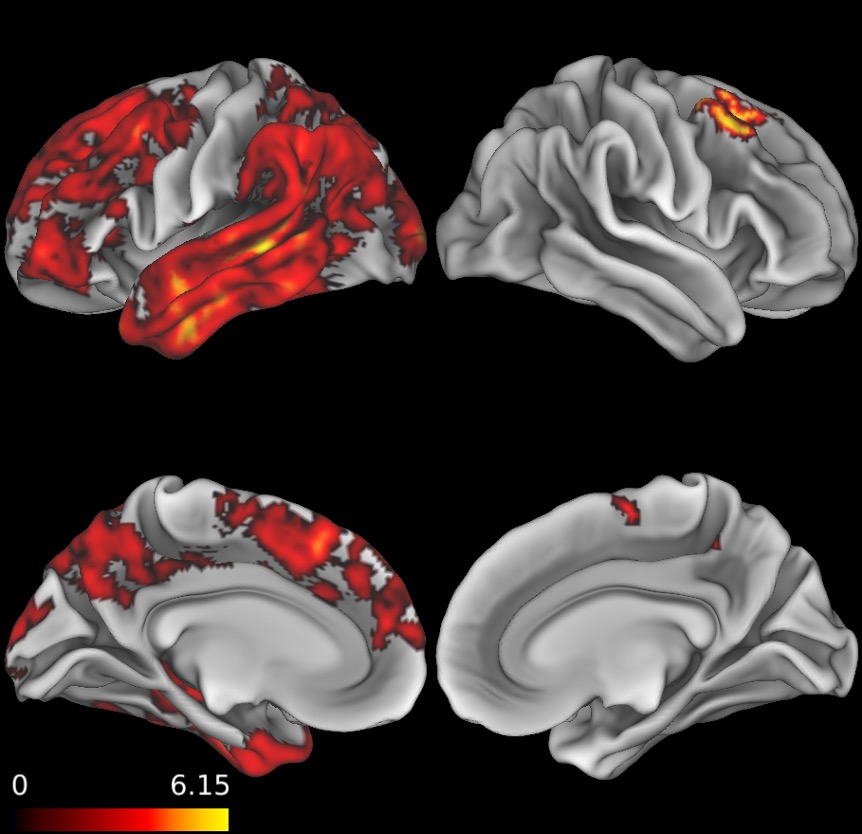

Supplement: Supplementary file 1 [file nol-1-4-474-s001.jpg]

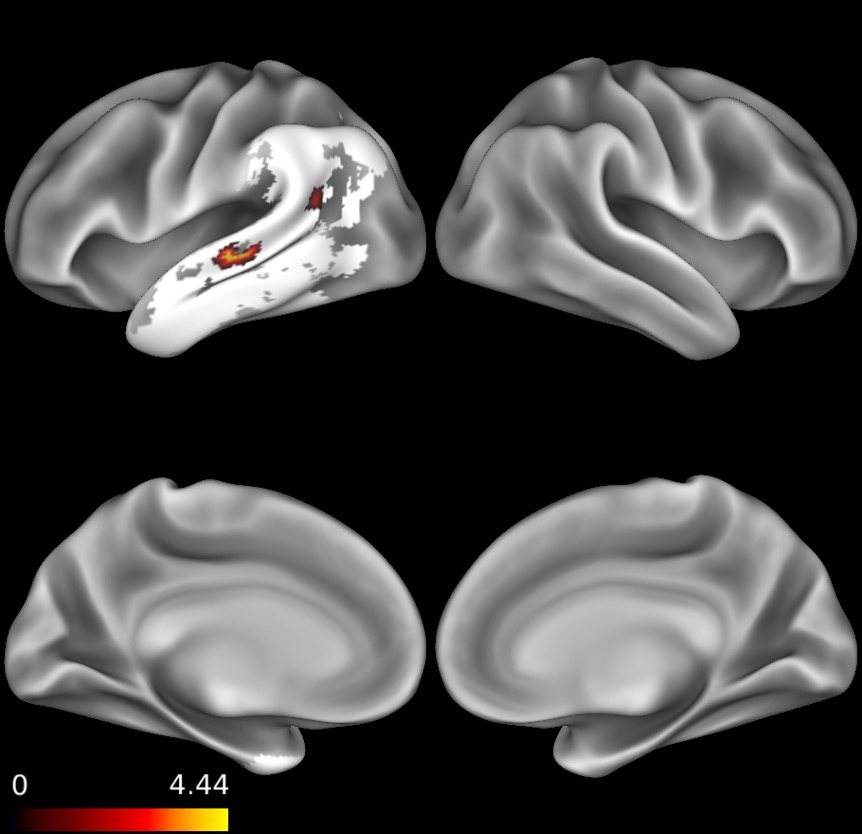

Supplement: Supplementary file 2 [file nol-1-4-474-s002.jpg]

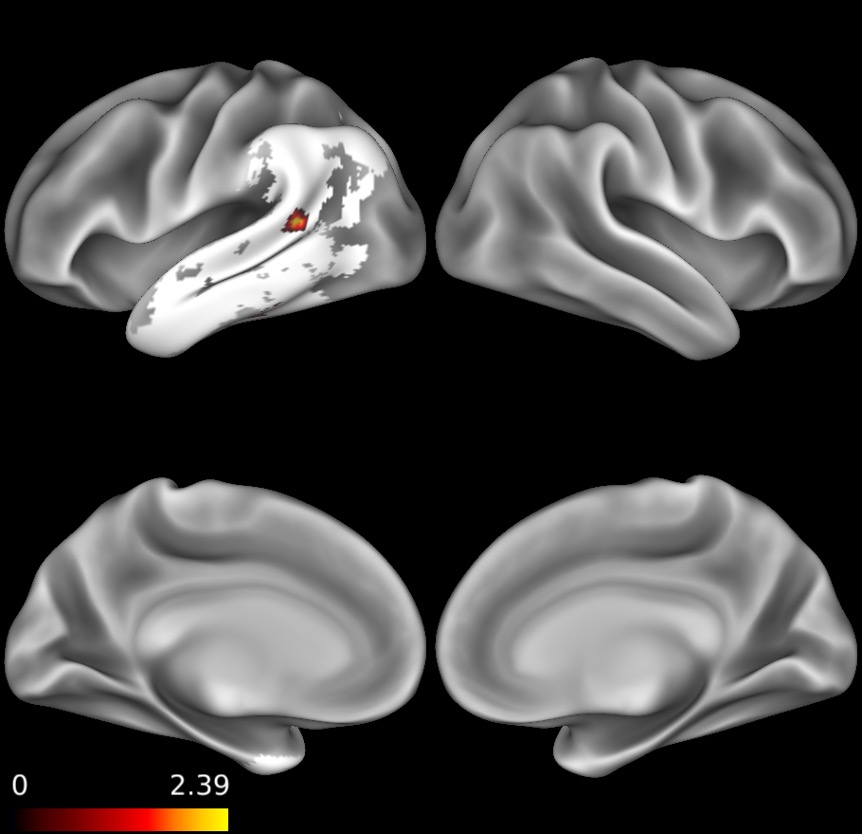

Supplement: Supplementary file 3 [file nol-1-4-474-s003.jpg]
